# A pilot survey of farm animal welfare in Serbia, a country preparing for EU accession

**DOI:** 10.1002/vms3.72

**Published:** 2017-10-23

**Authors:** Clare J. Phythian, Siobhan Mullan, Andrew Butterworth, Sarah Lambton, Jelena Ilić, Jelena Burazerović, Elvir Burazerović, Katharine A. Leach

**Affiliations:** ^1^ Animal Welfare and Behaviour Group School of Veterinary Sciences University of Bristol Langford Bristol BS40 5DU UK; ^2^ Faculty of Veterinary Medicine Section for Small Ruminant Research Norwegian University of Life Sciences 4325 Sandnes Norway; ^3^ Organisation for Respect and Care of Animals ‐ ORCA Risanska 1 11000 Belgrade Serbia

**Keywords:** animal welfare, farm animals, farmer perceptions, outcome measures, Serbia

## Abstract

The selection and field application of animal‐based welfare measures for pigs, sheep, dairy cows and broilers was the first step towards the future development of welfare improvement schemes for Serbia – a country that is applying for EU accession. The aim of this pilot study was to: (1) test the feasibility of a protocol for monitoring farm animal welfare in Serbia, (2) ascertain preliminary data on animal‐based outcomes of farm welfare and (3) gain insight into Serbian farmers’ understanding of animal welfare as part of a wider project working towards inclusion of animal‐based assessments in a ‘higher welfare’ voluntary assurance scheme. This study encompasses the first national survey of farm animal welfare in which animal‐based outcomes were tested on 105 farms by a total of ten trained assessors. Data on the views and aspirations of the farmers from these 105 farms were also systematically gathered during face‐to‐face interviews. Existing animal‐based measures for pigs, sheep, dairy cows and broilers that have been successfully applied and identified as valid, reliable and feasible measures in other countries, were found to be largely transferable. However, some on‐farm protocols, previously used in other countries, had to be shortened for logistical reasons when used in Serbia. Our findings suggest that further refinement may be needed in order to allow local application of all measures. While the term ‘animal welfare’ has only recently been introduced into the Serbian language, seventy‐three percent of farmers had heard of it. Overall, few positive associations were found between farmer satisfaction with animals’ living conditions and animal‐based data. Many farmers had aspirations to develop and expand their farms, which may potentially enhance animal welfare, but these farmers identified that financial and technical advice and support would be needed in order to achieve these goals.

## Introduction

Serbia is currently undergoing the process of accession for approval and entry as a member state of the European Union (EU), having applied for membership in December 2009, and confirmed as a candidate country in March 2012. An important priority in the accession pathway is the harmonization of animal welfare regulations (European Commission, 2007). Therefore, a key area for activity has been the adoption of the first ‘Animal Welfare Law ’ in Serbia in 2009. The National Strategy of Agriculture and Rural Development (2014–2024) recognizes animal welfare as an important aspect for development of Serbian agriculture and rural development. Farm animal welfare has also been made a significant part of the National Programme for Rural Development (NPRD) strategy for improvements in sustainable agriculture in Serbia, through initiatives aimed at strengthening administrative capacities for implementation of legislation, education of consumers and farmers, and training of government employees. The NPRD strategy has encouraged development of new products to fulfil the demands of contemporary markets, and recognized that enhanced animal welfare may help to develop new trading opportunities. However, the strategy has recognized a current limitation, in that many farmers in Serbia are unaware of current concepts of animal welfare and do not have adequate access to training (NPRD, 2011).

Other countries within Europe have also recently increased their emphasis on animal welfare in order to achieve EU accession. For example, Croatia has been working on aspects of its pig production systems, in relation to the EU pig welfare directives (Wellbrock *et al*. 2009) and Bulgaria has implemented legislation relating to animal welfare in order to satisfy EU directive 98/58/EC (Harizanova & Peneva 2009). Hungary includes standards for the welfare of farm animals in an Act on protection and humane treatment of animals, which satisfies the same EC Directive (Gudaj *et al*. 2012). During preparations for EU accession in the Former Yugoslav Republic of Macedonia, it was identified that some welfare legislation that exceeded EU requirements was already in place, while other aspects required amendment (Keeling *et al*. 2012). This indicates that current animal welfare standards in some accession countries may be sufficient for EU regulations, but some may not. In addition, EU membership offers the opportunity to exploit new market openings for animal products. Accordingly, this has led several Eastern European countries to raise the profile of farm animal welfare and to invest in specific training on animal welfare for veterinary surgeons and other professionals prior to, or subsequent to, EU accession. For example, animal welfare training courses for veterinarians in Hungary and Romania have been conducted under the auspices of the Federation of Veterinarians in Europe (FVE).

As part of an EU‐financed project ‘Farm Animal Welfare Standards in Serbia’ (FAWSS) a national pilot survey of the welfare of cattle, sheep, pigs and poultry on farms in Serbia was conducted. The ultimate aim was to work towards inclusion of animal‐based assessments in a ‘higher welfare’ voluntary assurance scheme. Prior to this project some animal welfare assessments of cattle had been made in Serbia based on the Animal Needs Index (ANI) (Bartussek *et al*. 2000), and the ANI with some additional features (Relic *et al*. 2010). A project entitled ‘Welfare and Biosecurity Standards – Development and Implementation in Improvement of Dairy and Pork Production’ was financed by the Ministry of Science and Technology Development of the Republic of Serbia (Hristov & Stanković 2009), which resulted in published data on some welfare criteria, including the use of the Welfare Quality® protocols for assessing the welfare of calves (Hristov *et al*. 2011a) and adult cows on three dairy farms (Hristov *et al*. 2011b). A small‐scale assessment of some selected measures from the Welfare Quality® protocol on six Serbian dairy farms was reported by Ostojić‐Andrić *et al*. (2011). Relic *et al*. (2010) believed that this work indicated an improvement in dairy cows’ welfare from 2006, but did not provide detailed evidence to support this claim. Therefore, to the authors’ knowledge, no national survey data on farm animal welfare animal‐based outcomes in Serbia currently exists.

The aim of this pilot project was to undertake the first national survey of the welfare of farm animals in Serbia to, firstly; test the feasibility of a protocol for monitoring farm animal welfare in Serbia, secondly; to provide preliminary data on farm animal welfare conditions using a set of animal‐based welfare indicators, and thirdly; to gain an understanding of farmers’ perception of the newly introduced ‘animal welfare’ term in the Serbian language (‘dobrobit zivotinja’ – translates approximately to ‘good being’). Our ambition was to gain an insight into changing perceptions – particularly important in a country where the overall concept of animal welfare, and farm animal welfare assessment schemes (including farm assurance schemes) that aim to promote and support animal welfare, are relatively new concepts (Relic *et al*. 2010).

## Materials and methods

A selection of potentially suitable animal‐based outcome measures was identified by a team of farm animal welfare scientists, building on these people's experience from previous UK and EU projects and combined with the findings of previous scientific studies assessing the reliability and feasibility of applying animal‐based welfare indicators of cattle (Welfare Quality®, 2009a), pigs (Welfare Quality®, 2009b; Mullan *et al*. 2011), poultry (Welfare Quality®, 2009c; Butterworth *et al*. 2013) and sheep (Phythian *et al*. 2012, 2013a). For each of the four farm animal species, body condition, cleanliness and integument lesions and lameness were considered to be key animal‐based outcome measures to be included in the on‐farm protocols. These measures were supported by some additional species‐specific outcome measures ‐ some examples; rectal prolapse rates (pigs), eye abnormalities (lambs). Information on key management descriptors was also obtained at the same time as the visit during face‐to‐face interviews with the participating farmers.

### Welfare assessment protocols

Animal welfare scientists from the University of Bristol School of Veterinary Sciences compiled an on‐farm assessment protocol for dairy cattle, pigs, broilers and sheep. The protocol was designed to be applicable in typical Serbian farm situations according to existing knowledge of management systems. The validity, reliability and feasibility of the animal‐based welfare indicators selected for these four farm animal species have been previously examined in other EU countries and are reported elsewhere (Garner *et al*. 2002; Leach *et al*. 2009; Mullan *et al*. 2011; Phythian *et al*. 2012). No invasive methods were used. All animal‐based outcome measures were scored according to species‐specific protocols and required close observation of individual animals. For broilers and sheep, some measures, such as body condition scoring, also required some low impact handling and palpation (manual examination). Table 1 summarizes the selection of animal‐based outcomes adopted, the methods of assessment, and suggested sample sizes for each species.

**Table 1:** Sample size recommendations, method of assessment and median and range of animal‐based outcome measures applied in the national pilot survey

Species and production stage	Suggested sample size	Animal‐based measure	*n* farms assessed	Median (range) farm percentage prevalences of outcome*_,_ †
Fattening pigs (FP) and sows (S)	Minimum 30 fattening pig pens and 50 sows	Oral behaviour (proportion of investigating pigs contacting enrichment)‡	11 S, 16 FP	0 (All 0) (S), 0 (0–82) (FP)
		Body lesions‡	16 S, 18 FP	16 (0–45) (S), 11 (0–39) (FP)
		Bursitis§		57 (0–100) (S), 70 (17–99) (FP)
		Lameness§		3 (0–4) (S), 1 (0–27) (FP)
		Manure on the body§		0 (0–100) (S), 40 (0–69) (FP)
		Body condition (thin)§		0 (0–38.2) (S), 0 (0–17) (FP)
		Skin condition§		0 (All 0) (S), 0 (0–4) (FP)
		Rectal prolapse§		0 (0–9) (S), 0 (All 0) (FP)
		Pumping (laboured breathing)§		0 (0–20) (S), 0 (0–2) (FP)
		Ruptures and hernias§		0 (All 0) (S), 0 (0–3) (FP)
Fattening pigs only	Minimum 30 fattening pig pens	Tail lesions§	18	0 (0–13)
		Twisted snouts§		0 (0–3)
Sows only	Minimum 50 sows	Body condition (thin)§	16	0 (0–38)
		Shoulder lesions‡		0 (0–13)
		Vulva lesions‡		1 (0–25)
		Uterine prolapse§		0 (0–20)
		Metritis§		0 (0–20)
		Mastitis§		0 (0–20)
Sheep	Minimum 50 sheep (includes ewes, rams and lambs 3 months‐old and over)	Body condition (thin)¶	21	Not assessed***
		Lameness**		4 (0–4)
		Eye abnormalities††		0 (0–34)
		Dull demeanour††		0 (0–4)
		Injuries and wounds††		0 (0–6)
		Skin lesions††		0 (0–13)
		Mastitis††		0 (0–29)* ^,^ ***
		Pruritis††		0 (0–13)
		Breech soiling (faeces)††		8 (0–100)
		Wool loss††		13 (0–53)
Lambs	Minimum 30 lambs (under 3 months old)	Demeanour/responsiveness‡‡	16	0 (0–100)
		Eye abnormalities‡‡		0 (0–25)
		Hunched posture‡‡		0 (All 0)
		Body condition (thin)‡‡		0 (All 0)
		Lameness‡‡		0 (0–25)
Dairy cattle	Maximum 30 cows	Body condition (thin)§§	28	5 (0–36)
		Dirty hindleg§§		28 (0–90)
		Dirty flank§§		19 (0–77)
		Dirty udder§§		13 (0–88)
		Swollen tarsus§§		0 (0–13)
		Swollen carpus§§		0 (0–27)
		Hairloss neck/shoulder/back§§		0 (0–17)
		Lame or severely lame (assessed moving)§§	20	3 (0–32)
		Lame (tied)§§	8	2 (0–25)
Broiler chickens	Minimum 100 broilers (close to slaughter age, approximately 34 days old)	Cleanliness¶¶	19	15 (0–60)
		Footpad dermatitis¶¶		24 (0–37)
		Hock burn¶¶		13 (0–20)
		Gait score¶¶		25 (0–51)

^*^Where a scoring system for pigs, sheep and cattle had three or more levels, the prevalence of any affected animals, regardless of severity, is given.^†^For broilers the outcome scores relate to the % of birds score 2 or more for cleanliness, footpad dermatitis and hock burn, and score 3 or more for gait score.^‡^Mullan *et al*. (2011).^§^Welfare Quality® (2009b).^¶^Russel (1984).^**^Phythian *et al*. (2012).^††^Phythian (2011).^‡‡^Phythian *et al*. (2013b).^§§^Welfare Quality® (2009a).^¶¶^Welfare Quality^®^ (2009c).^***^Farmers did not allow palpation/close examination to permit indicator assessment according to the protocol.

### Assessor recruitment and training

Ten assessors, consisting of four qualified veterinary surgeons, four undergraduate veterinary students and two agricultural studies graduates received a total of 8 days’ training (2 days’ intensive training per species), provided by species‐specific animal welfare scientists. Training involved 1 day of interactive discussions and seminars on animal‐based welfare measures and assessment protocols, and 1 day of on‐farm practical training. During classroom training, scoring exercises using images and videos were undertaken to promote standardization of outcome assessments. Assessors also carried out practice interviews with farmers to pilot test the questionnaire prior to the national survey. The on‐farm training for each of the species included visits to five farms to include one pig, one broiler, one sheep and two cattle farms. During on‐farm training, aspects of the protocol were demonstrated and trainees tested the application of the animal‐based measures under the guidance of the trainer. At the end of each training visit, the group of assessors discussed their individual assessment results to assist in alignment of scoring attributes. Following completion of all the farm training visits, the assessors also attended a 1‐day meeting in which they examined reference photographs and discussed scoring methods to encourage standardization, and to help reach scoring consensus (methodology as per Tuyttens *et al*. 2015). While the reliability of observer scoring assessments was informally explored and discussed during the interactive and practical training, the inter‐observer repeatability of assessors was not numerically quantified during this pilot due to logistical, financial and time constraints.

### Farm assessments

Livestock farms in Serbia are typically small ‐ most (77%) of holdings are less than 5 hectares (12.4 acres), and are usually mixed farms rearing a variety of livestock species (NPRD 2011; Official Gazette [Link]). The Serbian agricultural consensus of 2012 (Official Gazette [Link]) indicated that 908 000 cattle were managed on 177 000 farms (28% of total holdings), with an average of 5.1 cattle per farm. Around 70% of farms had one to two dairy cattle with an average of 2.8 dairy cows per farm. Just under a quarter (24.5%) of all farms managed the national flock of 1.73 million sheep with a mean of 11.2 animals per flock, and 3.4 million pigs were managed across 355,000 farms (56% of total holdings) with a mean number of 9.6 pigs per farm. Half of the 414 000 national poultry flock were broilers – the species managed at the highest intensity, with an average of 28 000 birds per farm (Official Gazette [Link]). Farms that were considered representative of Serbian management types and specializing in each of the four livestock species were selected from the database of farms provided by the Ministry of Agriculture, Water Management and Forestry of Serbia, Veterinary Office. Since informed consent was a requirement for farmer participation, convenience sampling was undertaken. Farms were individually contacted by local veterinary inspectors, to allow selection of a representative geographical distribution of farms and management type from across Serbia for each species. A range of farm sizes was included, excluding ‘subsistence farms’ where animal products were only for home consumption. As a result, 24 broiler, 20 fattening pig, 18 sow, 27 sheep and 28 dairy cattle units were recruited. Since subsistence farms were excluded from the recruitment pool for this survey, and farms specializing in one livestock species were preferentially recruited, the numbers of sheep, cattle and pigs per farm exceeded the mean 2012 census figures. The assessment protocols were used on a total of 105 farms during November and December 2011. On average, two farms per day were visited, by pairs of assessors, and each pair of assessors conducted 20 farm visits, covering all four species.

### Questionnaire

Questionnaire data collected from each farm included; animal numbers, general husbandry and productivity information, mortality records, routine use of mutilations (castration and tail docking), and bulk milk somatic cell count for dairy herds (as an indicator of udder health, since individual cow records were not usually available). The assessor conducted a short (approximately 15 min) semi‐structured face‐to‐face interview with the farmer. The same open and closed questions were used on all farms to ascertain background information relating to farmer understanding and perceptions of animal welfare. These questions were drafted by the Serbian partners. Translated into English, the questions were:

*Have you heard of animal welfare?*

If so,

*What does animal welfare mean to you?*

If not,

*What does animal care mean to you?*

All respondents were then asked:

*Are you satisfied with living conditions for your animals?*

*Is there anything you want to change (regarding animal living conditions)?*

*Do you need support for improving conditions on your farm?*

*If yes, what kind of support?*

*What are your plans for your farm?*

The questionnaires were conducted in Serbian, and the farmers’ responses were recorded on paper by the interviewer at the time of the interview.

### Data summary and analysis

Data from individual animal‐based outcome measures were used to provide a group‐level prevalence (percentage observed) for each of the recorded measures for each farm. The median and range for the measure values across all farms were determined.

The terms or words used by the farmers when responding to the questions on ‘What does animal welfare mean to you?’ and ‘What does animal care mean to you?’ were allocated to the following ‘term categories’ at the point of data entry: Good care, Good food, Good housing, Good environment, Good health, Good treatment, Good hygiene, Five freedoms, Better production. The features of their farms, or systems or aspects which farmers wanted to change, and the plans they had for the farm, were allocated to broad thematic categories by one researcher and checked by a second researcher following data entry and prior to analysis.

The proportions of farmers who had, and had not, heard of the term ‘animal welfare’ were calculated per species group. For those who ‘had’, the elements included in their description of what ‘animal welfare’ meant to them were categorized, and the proportion of farmers including each ‘category term’ was then calculated. This was repeated for those who ‘had not’ heard of animal welfare, but described welfare indirectly in terms of animal care (rather than animal welfare). Within each species, the herd or flock sizes of the farmers who ‘had’ and ‘had not’ heard of animal welfare were statistically examined. To test the hypothesis that the farmers’ degree of satisfaction with farm conditions might be related to animal outcome measures, a Mann–Whitney test was used to compare the prevalence of selected welfare outcomes between farms where farmers were and were not satisfied with their animals’ living conditions (in all cases where the number of unsatisfied farmers was greater than five). Significance testing was set as *P* = 0.05.

## Results

### Animal based welfare outcomes

The results of the key animal‐based welfare outcome assessments are summarized in Table 1. A short summary of the key points for each species follows:

#### Pigs

A total of 701 sows from 91 pens on 16 farms, and 3801 fattening pigs from 303 pens on 18 farms were assessed. Fattening pig farms had a mean of 1608 pigs and 18 pigs per pen, and farms with sows had a mean of 310 sows, 18.8 piglets per sow per year and a 33 day weaning age. Enrichment was observed on three fattening pig farms (chains or plastic tubes) and five sow farms (straw, wood, earth or toys). Thirty‐five percent (%) and 11% of fattening pig and sow farms respectively had slatted floors. Fattening pig farms had a mean of 100% of castrated male pigs and 95% of pigs were tail‐docked. Breeding farms had a mean of 94% of castrated male pigs, and 83% of piglets were tail‐docked and tooth‐clipped. Only one farm reported using anaesthesia for docking, castration and tooth clipping. There was an average annual mortality of 3.0% of sows on farms, and 0.3% of animals were culled. Overall 11% of farms reported cannibalism occurring in sow units. Fattening pig farms had an average annual mortality of 3.7% (range 0–10%) and 2.4% of fattening pigs were culled. Overall 15% of fattening pig farms observed cannibalism.

The most common lesion observed during the farm assessment visits was bursitis (swellings on the legs), with a median incidence of 57% (range 0–100%) of sows and 70% (range 17–99%) of fattening pigs per herd affected. No sows were observed interacting with any enrichment provided; however one fattening pig farm showed 83% of observed pigs with their mouth and snout in contact with the chain enrichment (as opposed to pen fittings) in contrast to the other two farms where the enrichment contact rate was reported to be <5%.

#### Sheep

A total of 1009 sheep, and 217 lambs were assessed on 27 farms in mountainous and hill (44%) and lowland areas (56%). Just over half (56%) of these meat‐producing sheep flocks were reportedly housed indoors year‐round, a further 11% were housed indoors with paddock access, and 33% of flocks were grazed (i.e. outdoor) throughout the production cycle. Just under 20% of flocks underwent seasonal migration to new pastures. Most (67%) flocks lambed indoors, 11% lambed outdoors, and the remaining 22% practised both. Tail docking, using a rubber ring or knife was reportedly practised by 63% of farms, and 19% routinely castrated approximately week‐old lambs by rubber ring (37%), burdizzo (26%), or surgical (37%) methods. Most sheep flocks (63%) were slaughtered at an abattoir, fewer (15%) performed home slaughter and the remaining 22% sold live sheep at markets. The pilot study period coincided with the lambing period, and with the housing of 20 out of 27 flocks. The most prevalent conditions observed were wool loss (median 13%, range 0–53%), followed by breech (faecal) soiling (median 8%, range 0–100%), and lameness (median 4%, range 0–4%). The recorded median prevalence of all young lamb indicators was zero (Table 1).

#### Cattle

A total of 776 cattle were assessed across 28 dairy herds. All were managed under intensive conventional management with the exception of one ‘calf‐at‐foot’ completely pasture‐based herd. Six herds had access to pasture, and 16 herds had access to outdoor space, for at least part of the year. Six herds were kept in tie‐stalls, 18 in loose housing, and four farms included both housing types. Straw was the most common type of bedding used. The number of cows per farm ranged from 7 to 683 with a median of 45. Reported median daily milk yield was 20 L (range 10–30 L). Annual average somatic cell count (SCC) data was available for 18 farms and values were relatively high, with a mean of 294 000 cells mL^−1^ (range 48 000–600 000). Four farms had an average SCC over 400 000. The average age of a cow in production was 5.25 years (range 4–7 years). Dirtiness was the parameter recorded with the widest range and highest prevalence by assessor pairs. Apparent prevalence ranged from 0 to 76% (median 19%) for dirty flanks, 0 to 90% (median 28%) for dirty hindlegs, and 0 to 87% (median 13%) for dirty udders.

#### Poultry

A total of 3620 birds from 24 broiler farms were individually assessed for mobility and 1807 for other individual bird assessment; 55% of flocks were Cobb genotype, 36% Ross, and the remaining 9% were Hubbard. The assessed farms had a mean of 24,182 birds per farm, and 11 396 (range 1500–45 000) birds per house. The average house size was 44 by 13 metres, giving a mean stocking density of 19.9 birds per m^2^. All were indoor intensive flocks, and none had outdoor access. Fifty‐four percent of flocks had straw litter, 13% were provided with wood shavings, and 29% had both types of litter. Thirty‐eight percent of flocks had some form of environmental enrichment; of those 78% were provided with straw bales and 11% (one flock) had perches. The mean mortality reported by farm staff was 3.1% per cycle, and morbidity was 2.0% per cycle. The mean mortality during the flock cycle, as recorded by assessors was 4.1%. On average 0.1% of the flock was culled during the flock cycle. The mean age for assessment in this study was 30 days, (approximately 13 days prior to average slaughter age), and a median of 25% (range 0–51%) of birds had a gait score of 3 (out of a maximum score of 5) or higher.

#### Questions on farmers’ views on animal welfare and aspirations for their farm

*Have you heard of animal welfare? What does animal welfare mean to you? or What does animal care mean to you?*

The majority of farmers (73%) had heard of animal welfare but there were some in each farm type group who had not. Only 17 sheep farmers (63%) had heard of animal welfare compared with 76% of each of the other groups of farmers (Table 2). The percentage of farmers who included multiple aspects of management and care in their definitions of welfare is shown in Table 2. Farmers who identified aspects of welfare were less inclined to include multiple explanatory concepts than those who had not heard of welfare and gave a description of animal care.

**Table 2:** Number (*n*) and percentage (%) of farmers including single and multiple aspects in their definitions of animal welfare (*n* = 62 farmers) or definitions of animal care, for those who had not heard of animal welfare (*n* = 21 farmers)

	Broiler		Cattle		Pig		Sheep	
	Animal welfare	Animal care	Animal welfare	Animal care	Animal Welfare	Animal care	Animal Welfare	Animal care
Percentage who had heard of welfare	76%		76%		76%		63%	
Number of farmers giving a single attribute (from those shown in Figs 1, 2)	9	2	11	0	12	1	7	3
Number of farmers giving multiple attributes (from those shown in Figs 1, 2)	7	3	9	6	7	5	10	7
Total responses	16	5	20	6	19	6	17	10
Percentage of farmers giving multiple factors	44%	60%	45%	100%	35%	82%	59%	70%

**Figure 1:**
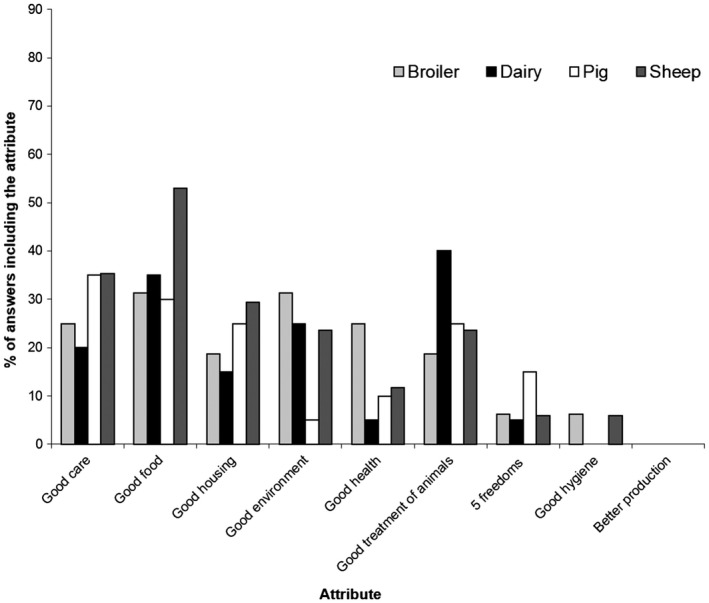
Components of farmers’ definitions of ‘animal welfare’ (*n* = 72 farmers).

**Figure 2:**
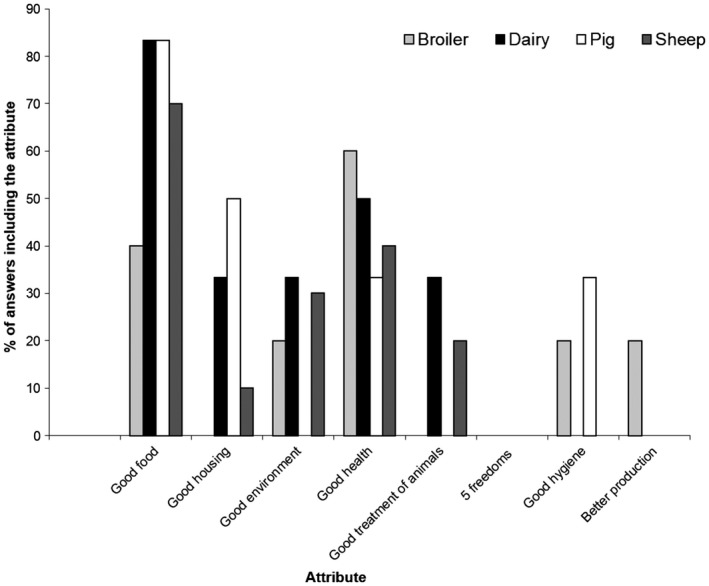
Components of farmers’ definitions of ‘animal care’ for farmers who had not heard of ‘animal welfare’ (*n* = 28 farmers).

‘Good food’ was the factor most commonly mentioned, either alone, or in conjunction with other factors, in explanations of both animal welfare (Fig. 1) and good care of animals (Fig. 2) (the latter for those who had not heard or animal welfare). ‘Good care and treatment’ were frequently included in definitions of good cattle welfare, while for broilers and pigs ‘good environment’ and ‘good health’ were also frequently mentioned. For sheep, care and housing were the next most commonly featured aspects in welfare definitions, after good food. Health was only mentioned twice in definitions of welfare by sheep farmers, but for those sheep farmers who had not heard of welfare, health was the next most frequently mentioned aspect of care, following good food. When considering the meaning of welfare, only one broiler farmer, four cattle farmers, two pig farmers and one sheep farmer mentioned the ‘Five Freedoms’ (FAWC – Farm Animal Welfare Council, 2009; Official Gazette [Link]).

*Are you satisfied with living conditions for your animals?*

*Is there anything you want to change (regarding animal living conditions)?*

Seventy‐four percent of farmers were satisfied with the living conditions of the animals on their farm (Table 3). The percentage of satisfaction was lowest for pigs and highest for broilers. Despite this, 70% of farmers stated they would like to change something about their farm. The types of changes described by the farmers as being desirable are summarized in Table 4.

**Table 3:** Number (*n*) and percentage (%) of farmers keeping different species of animals who were satisfied with their animals’ living conditions, and would like to make changes on the farm

	Broiler	Cattle	Pig	Sheep	Total
Are you satisfied with your animals’ living conditions? (*n* = 100)					
*n* Yes	18	20	17	19	74
*n* No	2	2	3	3	10
*n* Partially	2	4	5	5	16
% fully satisfied	82	77	68	70	74
Would you like to change something on the farm? (*n* = 98)					
*n* Yes	15	17	19	18	69
*n* No	6	9	5	9	29
% wishing to make a change	71	65	79	67	69
*n* farmers who answered ‘satisfied’ but would like to change something likely to improve welfare	13	11	12	16	52
% of farmers who answered ‘satisfied’ but would like to change something likely to improve welfare	72	55	71	84	53

**Table 4:** Number (*n*) and percentage (%) of farmers expressing a desire to change various aspects of their farm or animal husbandry

Type of change	Broilers *n* (%)	Cattle *n* (%)	Pigs *n* (%)	Sheep *n* (%)	Total *n*
Improved/new buildings	4 (19)	4 (15)	5 (19)	7 (26)	20
Ventilation	3 (14)		6 (23)		9
Slatted floor (new or replacement)			4 (15)		4
Decrease stocking rate in building	1 (5)	1 (4)			2
Automation of feeding/watering	2 (10)		4 (15)	1 (4)	7
Provide heating facility	2 (10)		1 (4)		3
Provide cooling facility	1 (5)				1
Medicine in water			1 (4)		1
Loose housing to replace tie‐stalls		3 (12)			3
Provide or increase outdoor access		5 (19)	1 (4)	1 (4)	7
Improve feeding	2 (10)	1 (4)		1 (4)	4
Bigger/better pasture				2 (7)	2
Better medical treatment		1 (4)		1 (4)	2
Total *n* farmers questioned	21	26	26	27	100

*Do you need support for improving conditions on your farm?*

*If yes, what kind of support?*

Eighty‐seven percent of farmers suggested they would need some support to improve their farm. The majority of these farmers reported that they needed financial support (76%), with the remainder needing both financial and expert technical support, or improved farm infrastructure. Twenty‐three percent said they would need expert advice either with or without financial support.

### Relationships between farmers' knowledge of the term ‘animal welfare’, or farmers' satisfaction with animals’ living conditions, and animal welfare outcome measures

The comparison of welfare measures on farms where the farmer had or had not heard of welfare showed no significant differences. A few measures demonstrated weak trends. For sows, there was a weak trend for a higher median prevalence of two indicators if the farmer had heard of welfare: this was for bursitis (70 vs. 25%, *P =* 0.17) and body lesions (23.3% vs. 0, *P =* 0.11). It is possible that there was confounding between prevalence and herd size, since there was a trend for farmers who had heard of animal welfare to have more pigs (75 vs. 37, *P =* 0.28). Farmers who had heard of welfare tended to have smaller flocks of sheep (30 vs. 50, *P =* 0.2). In this group there was a trend for fewer sheep with wool loss (7 vs. 17%, *P =* 0.15). Dairy herd size did not differ between farmers who had or had not heard of welfare (76 vs. 67, *P =* 0.92). A trend was observed for dirty flanks in cows, which appeared to be more prevalent where farmers had heard of the term ‘animal welfare’ (19 vs. 3%, *P =* 0.08).

Table 5 presents comparisons in herd/flock size, and the prevalence of selected measures (those with the highest prevalence), between the animals of farmers who were and were not satisfied with their animals’ living conditions. This analysis was carried out to investigate whether farmers’ opinion of satisfaction might be related to the welfare indicators for their animals i.e. would they be less satisfied if the animals demonstrated more indicators of poor welfare on their farms? For fattening pigs there was no significant difference in the numbers of pigs between satisfied and dissatisfied farmers, but when farmers were satisfied with conditions, unexpectedly, the prevalences of body lesions and lameness showed a trend to be higher (*P =* 0.07 and 0.10 respectively). For sows, statistical comparisons were not made as only five farmers were not satisfied with living conditions. However, a trend was observed in that the median prevalences of bursitis, body soiling and body lesions were numerically higher where farmers were dissatisfied.

**Table 5:** Median and range (in brackets) of herd/flock size and prevalence of selected animal welfare outcome measures on farms with different levels of farmer satisfaction with animals’ living conditions

Species	Satisfied	Not satisfied	Mann–Whitney *P* value
*Growing pigs*	*(n = 11)*	*(n = 7)*	
*n* pigs	212 (50–8500)	357 (50–14700)	0.93
Poor body condition	0 (0–17)	1 (0–5)	0.82
Bursitis	67 (23–98)	74 (17–99)	1.00
Manure on body	23 (4–55)	44 (0–70)	0.36
Body lesions	17 (0 ‐ 40)	7 (3–14)	0.07
Lame	3 (0–26)	0 (0–6)	0.10
Tail docked	100 (7–100)	100 (0–100)	0.59
*Sows*	*(n = 11)*	*(n = 5)*	
*n* pigs	41 (5–1200)	200 (6–2150)	
Poor body condition	0 (0–20)	0 (0–38)	*
Bursitis	38 (0–100)	99 (50–100)	
Soiled	0 (0–60)	42 (0–100)	
Body lesions	14 (0–32)	25 (8–48)	
Tail docked	100 (0–100)	100 (75–100)	
*Sheep*	*(n = 19)*	*(n = 8)*	
*n* sheep	61 (9–303)	120 (42–413)	0.10
Wool loss	11 (0–52)	16 (0–52)	0.60
*Cattle*	*(n = 20)*	*(n = 6)*	
*n* cattle	75 (7–7000)	67 (38–345)	0.46
Lameness	1.5 (0–29)	7.5 (0–32)	0.41
Dirty flank	13 (0–76)	21 (0–30)	1.00
Dirty legs	28 (0–90)	29 (0–57)	0.90
Dirty udder	13 (0–88)	13 (0–43)	0.98
Any tarsal abnormality†	0 (0–7)	4 (0–12)	0.07

^*^Mann–Whitney test not performed due to low sample size (*n* = 5).^†^Sum of hair loss, swelling and lesions of tarsus.

Farmers who were satisfied with conditions for their sheep tended to have smaller flocks (median flock size of 61 compared with 120 for satisfied farmers, *P =* 0.1). The only animal‐based sheep welfare measure with a high enough prevalence to be included in the analysis was wool loss. However, a significant association between the reported level of farmer satisfaction and the recorded prevalence of wool loss was not identified. In contrast, there were no significant differences in the dairy herd sizes between farmers who were and were not satisfied (75 vs. 67; *P =* 0.46). When farmers were satisfied, there was a trend for fewer tarsal abnormalities (0 vs. 4% hair loss, lesion or swelling, *P =* 0.07). As only four broiler farmers expressed any dissatisfaction with living conditions for their birds, no comparison of satisfaction levels was made for broiler farms.

### What are your plans for your farm?

When describing plans for the farm, many farmers mentioned the intention to increase production (Table 6). Only one dairy farmer and one pig farmer planned to decrease production. Several had aspirations to make alterations to the animals’ living conditions and buildings, such as improving ventilation or protection against cold or heat. A specific aim for one dairy farmer was to move from tethering to loose housing for the cows. Another planned to keep their sheep on pasture rather than housed indoors. On‐farm slaughter of livestock was also an aspiration for two broiler and two sheep farmers.

**Table 6:** Number (and percentage) of farmers mentioning particular plans for the future

Changes planned	Broilers	Cattle	Pigs	Sheep	Total
Increase production	9 (43)	8 (31)	4 (15)	20 (74)	51
Decrease production	0	1 (4)	1 (4)	0	2
New or improved buildings or equipment	4 (19)	7 (27)	4 (15)	6 (22)	21
On‐farm slaughter of livestock	2 (9)	0	0	2 (7)	4
Change breed	0	0	0	1	1
Total *n* farmers questioned	21	26	26	27	100

## Discussion

There is no doubt that the EU accession process has increased attention regarding the topic of animal welfare in Serbia (2011), as has occurred in other countries (Harizanova & Peneva 2009; Wellbrock *et al*. 2009; Keeling *et al*. 2012). This project has provided the opportunity to explore farmer attitudes as they move beyond the institution of law. Some of the fundamental steps towards the establishment of a framework for farm animal welfare standards for Serbia have been taken, and this may allow a position from which improvements in animal welfare can be encouraged. The steps undertaken by the project included piloting an approach for the selection of animal‐based outcome measures and training a group of farm animal welfare assessors. This pilot survey has also provided the largest data set so far recorded on animal‐based welfare measures for farm animal species in Serbia. The availability of such data from the Balkans region and neighbouring countries in general has been previously limited. Prior to this study, only four papers had reported the use of the full Welfare Quality protocol from the region, and were limited to cattle and poultry data (Vučemilo *et al*. 2012; Popescu *et al*. 2013; Prodanov & Ilieski 2013; Kirchner *et al*. 2014).

### Species‐specific results

Across all the four farm animal species examined, there was a mixed picture of animal welfare on Serbian farms. Some farms appeared to achieve relatively low levels of body lesions in pigs, which might have been achieved through good stockmanship in spite of apparent intensive production methods and observed deficiencies in housing. On others, specific management deficiencies were reflected in poor welfare standards, as demonstrated through a high prevalence of specific animal‐based outcomes. Some comparisons with other farms are available for dairy cattle in neighbouring regions, where the main animal welfare concern appeared to be cleanliness. The most comprehensive studies are available for Romania, where both Popescu *et al*. (2013) and Kirchner *et al*. (2014) carried out the full WelfareQuality^®^ protocol on 80 and 10 dairy farms respectively. Serbian results for the prevalence of thin cows were comparable to those reported by Popescu *et al*. (2013) but cows appeared to be cleaner in Serbia. This might be influenced by the fact that the farms included in this Serbian assessment included loose‐housed herds, while Popescu *et al*. (2013) only studied tied cows. However, Ostojić‐Andrić *et al*. (2011) considered the hygiene of both loose and tied dairy cows on six farms in Serbia to be inadequate. The prevalence of lameness in cows recorded in this project is very low in comparison with other surveys, for example, 3% vs. 31% reported in the Czech Republic by Sárova *et al*. (2011) using mobility scoring, and 1.7 vs. 15 or 26% when assessed in tiestalls, with and without regular exercise, respectively (Popescu *et al*. 2013). This might also have been influenced by sample size, causing underestimation in the larger Serbian herds.

For pigs, the small numbers of farms providing environmental enrichment led to the very low levels of enrichment‐directed oral behaviour observed on all except one fattening pig farm. This is an example of a situation where the survey highlighted an area where Serbian farms are not currently compliant with EU legislation. The lack of any environmental enrichment on 84% of finishing pig farms and 72% of sow farms would not be compliant with the EU Council Directive 2008/120/EC on the protection of pigs. This information will allow the Serbian government to focus resources on areas where changes are required to allow harmonization, in anticipation of EU accession.

Although many pig farmers wanted to change things about their farms, they appeared relatively content with the lack of environmental enrichment. If enrichment was provided in the form of straw or other soft bedding material this might also aid pig comfort as well as providing an outlet for exploratory behaviour, with likely reductions in tail and body lesions on some farms. To improve animal welfare, the motivations and attitude of farmers and their needs and perceived barriers for change need to be better understood. For example, if farmers believe an enrichment material such as straw is not important for pig welfare, or could be detrimental to pig health or productivity, or if building design is not compatible with straw provision, it is unlikely to be provided to pigs without clear legislative guidance and enforcement.

Broiler farms were perhaps most similar in character to those in EU member countries. However, assessments were made at an earlier age than recommended in the protocol (13 days on average before slaughter), especially given the slightly older slaughter age of broiler birds examined in other EU countries (Tuyttens *et al*. 2014). This presents difficulties for cross‐study comparison of specific animal welfare outcome results for gait score, foot pad dermatitis and hock burn. However, even at this younger age, there were high levels of footpad sores (pododermatitis) and lameness found in some of the Serbian broiler farms visited. The timing of assessments in this pilot study was to some extent determined by participating farmers. In future, animal‐based welfare outcome assessments might be scheduled to coincide with key periods in the production cycle.

Wool loss was the most prevalent condition found in Serbian sheep, but no similar sheep studies in the Balkans were found for data comparison. Further individual animal examinations and potential diagnostic tests would be required to determine whether wool loss was physiological and non‐pathological or whether it was associated with an underlying disease or environmental issue. Animal welfare assessments of sheep performed by observation from a distance, can present challenges for the detection of small skin lesions due to masking by the fleece (Napolitano *et al*., 2009). Given the lack of individual animal handling required for optimal assessment, it is likely that the level of skin and integument conditions in Serbian sheep flocks may have been under‐reported in this pilot survey.

### Feasibility of animal‐based outcomes

Overall, the objective of testing the feasibility of welfare assessment protocols, under Serbian conditions, for the chosen species, was achieved. Existing animal‐based welfare assessment indicators for pigs, sheep, dairy cows and broilers that have been successfully applied and identified as valid, reliable and feasible measures in other countries, were found to be largely transferable, but on‐farm assessment protocols for Serbia had to be shortened for logistical reasons. Further refinements to the protocols are also likely to be necessary

There were reported difficulties in carrying out assessments on some farms, particularly with sheep. According to the protocol, some sheep welfare outcomes required minimal restraint and gentle handling to facilitate assessment. With the exception of broilers and sheep, animal welfare assessments of other farm animals did not require handling to assess body condition. Body condition scoring of sheep specifically requires palpation of the lumbar vertebrae due to masking of condition by the fleece (Russel 1984). Despite specific and detailed practical training in this respect, an unexpected occurrence was that assessors did not assess body condition as instructed and fully described in the protocol. It was reported that farmers did not allow assessors to handle or gently restrain pregnant ewes. Accordingly, body condition scores (BCS) of sheep were not analysed further, and the results should be carefully interpreted in the light of the assessment method used.

There can be difficulties in gaining close contact with, and handling, sheep at certain periods. Gavojdian *et al*. (2011), examined other alternatives and assessed Romanian sheep using a combination of management questionnaires, producer disease records and measures of productivity. However, reliance on producer reports and a lack of an animal‐based focus risks missing serious and early lesions, such as sheep scab (*Psoroptes ovis*), which is an important and relevant welfare concern for Serbian sheep flocks. Given the importance of body condition ‐ globally accepted as a key welfare outcome for sheep including extensively‐managed animals (Morgan‐Davies *et al*. 2008; Phythian 2011; Phythian *et al*. 2011), improved communication with farmers ahead of visits to discuss available facilities and handling aspects may improve adherence to the protocol.

Further examination of the feasibility of assessing housed and pastoral animals managed under the resources and conditions found in Serbia and elsewhere may be useful. Altering the time of the assessment away from the immediate lambing period might make handling feasible on some farms, but for other flocks this might mean that the sheep would not be housed and therefore might present other practical challenges with the need to gather extensively‐managed animals. Pregnancy and lambing present specific animal welfare risks, and it is important that these periods are not disregarded. Future assessments could be timed to coincide with gathering for management and key production periods. Practical on‐farm solutions to handling animals, combined with improved communication ahead of visits may facilitate improved local application of all measures. Training and extension programs to engage and better inform farmers of the practical and economic value of outcome results (Main *et al*. 2012) and understanding of the relationship between animal health, welfare and management inputs would also be beneficial.

New or improved buildings were the most common desired improvement for sheep farmers, and related improvements in flock management could facilitate handling and examination of individual animals. Where suitable facilities are lacking, assessors could use portable and lightweight gates to create a mobile assessment pen. However, the issue of assessing grazing sheep welfare is not unique to Serbia and therefore, a group‐based method for some indicators (Phythian *et al*. 2012) was demonstrated to facilitate assessment of pastoral flocks.

### Farmer aspirations

Approximately a quarter of participants had not heard of the term “dobrobit zivotinja”, recently introduced into the Serbian language to convey the meaning of “animal welfare”. Greater challenges of understanding might be expected if these pilot interviews were extended to include the wider population; governments and non‐governmental organisations (NGO's) should be aware of this. Immink *et al*. (2010) mapped seven European countries according to how consumers, stakeholders and producers perceived the level and importance of farm animal welfare. The lowest ranking countries were Poland (newest member state involved) and Former Yugoslav Republic of Macedonia (preparing for accession). It is a ‘positive’ finding that nearly 75% of farmers had heard of the term ‘dobrobit zivotinja’ after its fairly recent introduction to the language, as part of the new Animal Welfare Law introduced in 2009. However, this finding also indicates clear potential for the ongoing and further animal welfare educational programs and improving farmer understanding and engagement in animal welfare in this region.

The relatively limited number of components of the Serbian farmers’ concept of animal welfare, which tended to be based on physical conditions rather than mental state, might be expected from farmers facing economic difficulties and without experience of value‐enhanced welfare‐based products (Bock & van Huik 2007; Kling‐Eveillard *et al*. 2007; Cziszter *et al*. 2011). As the farmers’ satisfaction with their animals’ living conditions did not show a clear relationship with the results of the welfare assessment, there may be challenges in initiating change in some situations where improvement is needed. However, the overlap between components of ‘animal welfare’ and ‘animal care’ in farmers’ definitions provides a good starting point for communications and a basis for producer educational campaigns.

Although many farmers had aspirations for farm alterations likely to benefit welfare, a challenge which should not be overlooked, is that 82% stated that they would require financial assistance to make changes to their farms. Relic *et al*. (2010) pointed out that Serbian farmers are hampered by outdated buildings, historical difficulties with long‐term planning, and financial constraints. Bulgarian farmers considered that welfare improvements would have a net cost, and were unwilling to make expenditure in the absence of a premium for products (Hristov & Stanković 2009; Tudorache *et al*. 2014). Tudorache *et al*. (2014) calculated that the costs of broiler production increased with the introduction of welfare laws in Romania. Interventions involving changes to husbandry and work practices may be the most feasible and practical changes which could be made in the face of financial constraints (Pritchard *et al*. 2012).

### Challenges, limitations and opportunities

It is recognized that there are limitations to the pilot data, in view of the lack of proven inter‐observer reliability, and the relatively small, self‐selecting sample of farms. However, this data, and its analysis, are of great potential value as a starting point for stakeholders in the Balkans, including legislators and state planning authorities, and informing debates as to where further resources for farm animal welfare, education tools and advisory actions may be relevant.

This pilot project created ten trained farm animal welfare assessors with increased experience and capacity in farm animal welfare assessment. The number of farms (*n* = 105) examined was based on feasibility, given the timing and funding restrictions, the logistics of visiting farms, and the stated aim to collect preliminary information regarding the four main types of livestock farms. Therefore the findings do not claim to give a detailed picture of the precise welfare state in Serbia, but rather, should be viewed as a preliminary informative data set on which to build, and a necessary starting point in the process of moving towards farm animal assurance schemes. It is clear that for future developments and training, standardization of practices must be firmly established, and evaluation of observer agreement should be prioritized.

The apparent low or zero median prevalence of several indicators may represent the true health and welfare status of these animals or reflect problems with assessment conditions or the ability to perform assessments fully. The low prevalence phenomenon is familiar to animal health and welfare researchers. Studies performed outside Serbia have identified similar issues with low levels of specific lesions on participating farms (Phythian *et al*. 2013a). Inevitably, with a voluntary project, recruitment of consenting participants may have biased the sample in favour of those with a greater interest in animal welfare and/or different, perhaps higher, welfare standards.

The prevalence of lameness recorded in all species was lower than has been reported in the majority of other studies. For example, in dairy cattle, researchers reported 31% prevalence in Czech herds (Popescu *et al*. 2014) and 32 and 20% in two different tiestall systems in Romania (Sárova *et al*. 2011). It is possible that the Serbian assessors, with limited training, were less likely to detect lameness than experienced researchers (Leach *et al*. 2010). The Serbian assessors were possibly closer in their sensitivity to farmers, who have often been reported to record a lower prevalence of lameness than researchers (Leach *et al*. 2010; Sárova *et al*. 2011).

Ideally, the inter‐observer reliability (IOR) of all assessors would have been tested prior to their pilot application in Serbia, but this was not feasible given the study resources. However, the issue of testing IOR is not uncommon to other animal welfare studies that have undertaken national or regional surveys or during the preliminary steps towards the development of farm animal welfare standards. The approach taken here is consistent with that of other researchers who did not test IOR but instead paid particular attention to the detailed training of assessors in order to reach a consensus and agreed standardization in observer scores ahead of on‐farm assessments (Tuyttens *et al*. 2014). This also mirrors the situation for farm assessors in voluntary or farm certification schemes who are trained to apply pre‐tested valid, reliable and feasible indicators contained in on‐farm assessment protocols, such as WelfareQuality®. The animal‐based indicators included for cattle, poultry and pigs reflect those used in the EU WelfareQuality® protocols and, as per those guidelines, detailed training of assessors in species‐specific protocols was a key aspect of this pilot project.

Despite this, Mullan (2011) identified that initial assessor agreement can be quite poor following welfare outcome training. It is possible that the assessors did not follow the protocols exactly, or were not able to get a good opportunity to observe cows and sheep walking, due to restrictions of the housing systems, or unwillingness of farmers to have visitors interacting with their animals (as with the handling for BCS in sheep), although this was not formally recorded. The conditions surrounding assessment may also have affected the ability to perform some welfare assessments. Sheep lameness levels (median 4%) were similar to the mean of 3.75% identified in Italian flocks (Caroprese *et al*. 2009) but were lower than in English and Welsh flocks (mean 7.1%) (Phythian *et al*. 2013b). Assessors reported they could not freely walk around sheep to assess their gait or assess lying animals; on many farms sheep gait assessment was performed in housed ewes akin to the group observation method, whereby assessments are made from a distance (Phythian *et al*. 2012). Therefore, the level of sheep lameness reported here is likely to represent the more severe cases.

A particular challenge, not unique to this project, was the time available for training and assessment visits. This was addressed by limiting the number of measures, and the sample size (particularly so for cattle). It is likely that the protocols adopted in this study would similarly need to be reduced in time if they are to be incorporated into Serbian farm animal welfare standards schemes. Animal welfare assessments in existing farm assurance schemes in the UK have usually been limited to 20–30 min due to time and financial constraints (Main & Mullan 2012). Other groups have investigated the effect of shortening the Welfare Quality® protocols (Heath *et al*. 2014; De Jong *et al*. 2016), although Radeski *et al*. (2015) recommended use of the full cattle protocol in the Former Yugoslav Republic of Macedonia. The protocols used in this project were adapted to the shorter time available and provided a useful range of information (data) in all species, but would be unsuitable for deriving the integrated Welfare Quality® criterion‐, principle‐ or overall‐scores.

Since this project was delivered, the Serbian Government has reviewed the project results and has included a short period (30 min) for animal‐based assessments within their program of annual statutory farm inspections conducted by government veterinary surgeons. While there remain challenges, this provides an example of how an accession country can go beyond base legislative requirements and common practices for animal welfare in the EU, by utilizing the infrastructure already in place for farm inspections – in this case official regulatory inspections, rather than relying solely on private voluntary assurance schemes.

### Implications for animal welfare

Introducing assessment frameworks for the first time in a country undergoing EU accession can stimulate positive actions and training processes directed towards improvements in animal welfare. For EU accession countries, this process of conducting a survey of the welfare of farm animals may be of real value to highlight areas for focused or supported welfare improvement and to assist in achieving EU legislation compliance. It may also serve to raise awareness of welfare assessment among farmers, increase capacity and experience in assessment methods, and increase understanding of regulatory and voluntary mechanisms for welfare improvement among stakeholders. This pilot project has already expanded the sphere of influence with regard to animal welfare in Serbia, by working directly with veterinary surgeons, students, agricultural graduates and farmers, involving them practically in assessments and discussions of direct indicators of welfare. Training has increased the pool of skills in animal‐based assessment. However, there remain clear challenges for trainers, assessors, producers and policy‐makers due to the limited resources and support infrastructure that are currently available.

## Conclusion

As a pilot study, this first national survey has captured some preliminary, baseline data for setting improvement targets. Pilot study results and experiences suggest that there are areas where further species‐specific training, and improved knowledge and competencies of assessors and farmers in identifying animal health and welfare conditions could inform improvements in farm animal welfare standards. Improved communication and planning of visits for optimal timing, and closer engagement of farmers in animal‐based assessments, could further facilitate adherence to and feasibility of the described protocols and animal handling. While ‘animal welfare’ (‘dobrobit zivotinja’) has only recently been introduced into the Serbian language, seventy‐three percent of farmers had heard of this term. Among those who have heard of animal welfare, there is scope to expand their understanding of what ‘welfare’ incorporates, although it is encouraging that many farmers described ‘welfare’ in positive terms. Many farmers (70%) reported they would like to make changes to their farms, many of which could be beneficial to animal welfare. Expansion, modernization and structural alterations to farms may offer animal welfare benefits, and this study may assist in planning for this due to more refined information on farmer and animal needs. This study supports the view that opportunities for welfare improvement exist, if technical advice and financial support are available, to facilitate change and progress the improvement aspirations of these farmers.

## Source of funding

This project was part of an EU‐financed project “Farm Animal Welfare Standards in Serbia” (FAWSS). The project was contracted by the Austrian Development Agency from the combined funds of the IPA 2011 programme, the Republic of Austria's assistance programme and Serbian national co‐financing (grant number 526‐00/2011/Grant69).

## Conflicts of interest

The authors declare no conflict of interest.

## Ethics statement

The authors confirm that the ethical policies of the journal, as noted on the journal's author guidelines page, have been adhered to. This on‐farm study involved non‐invasive measures and minimal handling of animals managed on commercial farms, as per routine stockperson assessments and veterinary physical examinations. The study received approval from the Veterinary Office of the Ministry of Agriculture, Forestry and Water Management of Serbia.

## References

[vms372-bib-0001] Animal Welfare Law Official Gazette of the Republic of Serbia, number 41/09; 2009.

[vms372-bib-0002] National Programme for Rural Development 2011–2013 Official Gazette of the Republic of Serbia 15/2011; 2011.

[vms372-bib-0003] Strategy for agriculture and rural development in Republic of Serbia for the period 2014–2020. Official Gazette RS nr. 85/2014.

[vms372-bib-0004] Bartussek H. , Leeb C. & Held S. (2000) Animal Needs Index for Cattle, ANI 35 L/2000. Federal Research Institute for Agriculture in Alpine Regions BAL: Gumpenstein, Austria.

[vms372-bib-0005] Bock B.B. & van Huik M.M. (2007) Animal welfare: the attitudes and behaviour of European pig farmers. British Food Journal 109, 931–944.

[vms372-bib-0006] Butterworth A. , Blokhuis H.J. , Jones B. , Veissier I. (2013) Relevance and Implementation of the Welfare Quality^®^ Assessment Systems. Improving Farm Animal Welfare: Science and Society Working Together: The Welfare Quality^®^ Approach. Wageningen Academic Publishers: Wageningen.

[vms372-bib-0007] Caroprese M. , Casamassima D. , Pier S. , Rassu G. , Napolitano F. & Sevi A. (2009) Monitoring the on‐farm welfare of sheep and goats. Italian Journal of Animal Science 8, 343–354.

[vms372-bib-0008] Cziszter L.T. , Acatincai S. , Sossidou E.N. , Szucs E. , Gavojidian D. , Tripon I. *et al* (2011) General knowledge of the Romanian farmers about the farm animal welfare. Lucrări ştiinţifice Seria Zootehnie 55. Universitatea de Ştiinţe Agricole şi Medecină Veterinară Iaşi: Romania.

[vms372-bib-0009] De Jong I.C. , Hindle V.A. , Butterworth A. , Engel B. , Ferrari P. , Gunnink H. *et al* (2016) Simplifying the Welfare Quality ^®^ assessment protocol for broiler chicken welfare. Animal 10, 117–127. 26306882 10.1017/S1751731115001706

[vms372-bib-0010] European Commission (2007) Commission Opinion on Serbia's application for membership of the European Union. European Commission: Brussels, Belgium.

[vms372-bib-0011] FAWC ‐ Farm Animal Welfare Council (2009) Available at: http://www.fawc.org.uk/freedoms.htm [Accessed 24 June 2014].

[vms372-bib-0012] Garner J.P. , Falcone C. , Wakenell P. , Martin M. & Mench J.A. (2002) Reliability and validity of a modified gait scoring system and its use in assessing tibial dyschondroplasia in broilers. Poultry Science 43, 355–363. 10.1080/00071660120103620 12195794

[vms372-bib-0013] Gavojdian D. , Pacala N. , Sauer M. , Padeanu I. & Voia S.O. (2011) Sheep welfare analysis in private farms from Timis county. Lucrări Ştiinţifice ‐ Universitatea de Ştiinţe Agricole şi Medicină Veterinară, Seria Zootehnie. 56, 132–136.

[vms372-bib-0014] Gudaj R.T. , Brydyl E. , Lehoczky J. & Komlósi I. (2012) Study of animal welfare status in dairy cow herds in Hungary. Biotechnology in Animal Husbandry 28, 509–516.

[vms372-bib-0015] Harizanova T. , Peneva M. (2009) Animal welfare and economic effectiveness in Bulgaria and EU farms. Lucrări ştiinţifice Zootehnie şi Biotehnologii 42, Timişoara.

[vms372-bib-0016] Heath C.A.E. , Browne W.J. , Mullan S. & Main D.C.J. (2014) Navigating the iceberg: reducing the number of parameters within the Welfare Quality ^®^ assessment protocol for dairy cows. Animal 8, 1978–1986. 25159607 10.1017/S1751731114002018

[vms372-bib-0017] Hristov S. & Stanković B. (2009) Welfare and biosecurity indicators evaluation in dairy production. Biotechnology in Animal Husbandry 25, 623–630.

[vms372-bib-0018] Hristov S. , Stanković B. , Todorović‐Joksimović M. , Mekić C. , Zlatanović Z. , Ostojić‐Andrić D. & Maksimović N. (2011a) Welfare problems in dairy calves. Biotechnology in Animal Husbandry 27, 1417–1424.

[vms372-bib-0019] Hristov S. , Zlatanović Z. , Stanković B. , Ostojić‐Andrić D. , Davidović V. , Joksimović‐Todorović M. *et al* (2011b) Dairy cows welfare assessment in loose stalls. Veterinarski Glasnik 65, 399–408.

[vms372-bib-0020] Immink V. , Ingenbleek P. , Keeling L.J. (2010) Report on development of policy instruments towards the Action Plan on Animal Welfare, SWOT‐analysis of policy instruments following brainstorm meeting and literature. Deliverable 3.1 EconWelfare Project.

[vms372-bib-0021] Keeling L.J. , Immink V. , Hubbard C. , Garrod G. , Edwards S.A. & Ingenbleek P. (2012) Designing animal welfare policies and monitoring progress. Animal Welfare 21(Suppl. 1), 95–105.

[vms372-bib-0022] Kirchner M.K. , Ferris C.P. , Abecia L. & Winckler C. (2014) Welfare state of dairy cows in three European low‐input and organic systems In: Proceedings of the 4th ISOFAR Scientific Conference (eds Rahmann G. & Arksoy U.), pp 33–36. 13‐15 October, Istanbul, Turkey. Johann Heinrich von Thünen‐Institut: Braunschweig, Germany.

[vms372-bib-0023] Kling‐Eveillard F. , Dockes A.‐C. & Souquet C. (2007) Attitudes of French pig farmers towards animal welfare. British Food Journal 109, 859–869.

[vms372-bib-0024] Leach K.A. , Dippel S. , Huber J. , March S. , Winckler C. & Whay H.R. (2009) Assessing lameness in cows kept in tie‐stalls. Journal of Dairy Science 92, 1567–1574. 19307637 10.3168/jds.2008-1648

[vms372-bib-0025] Leach K.A. , Whay H.R. , Maggs C.M. , Barker Z.E. , Paul E.S. , Bell A.K. & Main D.C.J. (2010) Working towards a reduction in cattle lameness: 1. Understanding barriers to lameness control on dairy farms. Research in Veterinary Science 89, 311–317. 20363487 10.1016/j.rvsc.2010.02.014

[vms372-bib-0026] Main D.C.J. & Mullan S.M. (2012) Economic, education, encouragement and enforcement influences within farm assurance schemes. Animal Welfare 21, 107–111.

[vms372-bib-0027] Main D.C.J. , Mullan S.M. , Atkinson C. , Bond A. , Cooper M. , Fraser A. & Browne W.J. (2012) Welfare outcome assessments in laying hen farm assurance schemes. Animal Welfare 21, 389–396.

[vms372-bib-0029] Morgan‐Davies C. , Waterhouse A. , Pollock M.L. & Milner J.M. (2008) Body condition score as an indicator of ewe survival under extensive conditions. Animal Welfare 17, 71–77.

[vms372-bib-0030] Mullan S. (2011) A pilot investigation of Farm Assurance assessors’ attitude to farm animal welfare as a confounding factor to training in pig welfare outcome measures. Animal Welfare 20, 413–421.

[vms372-bib-0031] Mullan S. , Edwards S.A. , Butterworth A. , Whay H.R. & Main D.C. (2011) Inter‐observer reliability testing of pig welfare outcome measures proposed for inclusion within farm assurance schemes. The Veterinary Journal 190, 100–109. 10.1016/j.tvjl.2011.01.012 21377385

[vms372-bib-0032] Napolitano F. , De Rosa G. , Ferrante V. , Grasso F. & Bragheri A. (2009) Monitoring the welfare of sheep in organic and conventional farms using an ANI 35 L derived method. Small Ruminant Research 83, 49–57.

[vms372-bib-0033] Ostojić‐Andrić D. , Hristov S. , Novaković Z. , Pantelić V. , Petrović M.M. , Zlatanović Z. & Nikšić D. (2011) Dairy cows welfare quality in loose vs tie housing system. Biotechnology in Animal Husbandry 27, 975–984.

[vms372-bib-0034] Phythian C.J. (2011) Development of indicators for the on‐farm assessment of sheep welfare. PhD Thesis, University of Liverpool, UK.

[vms372-bib-0035] Phythian C.J. , Michalopoulou E. , Jones P.H. , Winter A.C. , Clarkson M.J. , Stubbings L.A. *et al* (2011) Validating indicators of sheep welfare through a consensus of expert opinion. Animal 5, 943–952. 22440034 10.1017/S1751731110002594

[vms372-bib-0036] Phythian C.J. , Toft N. , Cripps P.J. , Michalopoulou E. , Winter A.C. , Jones P.H. *et al* (2012) Reliability of indicators of sheep welfare assessed by a group observation method. The Veterinary Journal 193, 257–263. 22266020 10.1016/j.tvjl.2011.12.006

[vms372-bib-0037] Phythian C.J. , Toft N. , Cripps P.J. , Michalopoulou E. , Winter A.C. , Jones P.H. *et al* (2013a) Inter‐observer agreement, diagnostic sensitivity and specificity of animal‐based indicators of young lamb welfare. Animal 7, 1182–1190. 23561038 10.1017/S1751731113000487

[vms372-bib-0038] Phythian C.J. , Cripps P.C. , Grove‐White D. , Jones P.H. , Michalopoulou E. & Duncan J.S. (2013b) Observing lame sheep: evaluating test agreement between group‐level and individual animal methods of assessment. Animal Welfare 22, 417–422.

[vms372-bib-0039] Popescu S. , Borda C. , Diugan E.A. , Spinu M. , Groza I.S. & Sandru C.D. (2013) Dairy cows welfare quality in tie‐stall housing system with or without access to exercise. Acta Veterinaria Scandinavica 55, 43. 23724804 10.1186/1751-0147-55-43 PMC3674972

[vms372-bib-0040] Popescu S. , Borda C. , Diugan E.A. , Niculae M. , Stefan R. & Sandru C.D. (2014) The effect of the housing system on the welfare quality of dairy cows. Italian Journal of Animal Science 13, 2940.

[vms372-bib-0041] Pritchard J.C. , van Dijk L. , Ali M. & Pradhan S.K. (2012) Non‐economic incentives to improve animal welfare: positive competition as a driver for change among owners of draught and pack animals in India. Animal Welfare 21, 25–32.

[vms372-bib-0042] Prodanov M. , Ilieski V. (2013) Welfare quality protocol provides an insight in the welfare problems of the laying hens in Republic of Macedonia. Proceedings, Days of Veterinary Medicine 2013, 4th International Scientific Meeting, 6‐8 September, Struga, Macedonia. p. 65.

[vms372-bib-0043] Radeski M. , Janevski A. & Ilieski V. (2015) Screening of selected indicators of dairy cattle welfare in Macedonia. Macedonian Veterinary Review 38, 43–51.

[vms372-bib-0044] Relic R. , Hristov S. & Bojkovski J. (2010) Application of methods to assess the welfare of dairy cows on farms in Serbia. Bulletin of University of Agricultural Sciences and Veterinary Medicine Cluj‐Napoca. Veterinary Medicine 67, 256–262.

[vms372-bib-0045] Russel A. (1984) Body condition scoring of sheep. In Practice 6, 91–93. 6735487 10.1136/inpract.6.3.91

[vms372-bib-0046] Sárova R. , Stéhulo I. , Kratinová P. , Firla P. & Śpinka M. (2011) Farm managers underestimate lameness prevalence in Czech dairy herds. Animal Welfare 2, 201–204.

[vms372-bib-0047] Tudorache M. , Custură I. , Van I. , Micloşanu E.P. & Vidu L. (2014) Influence of applying broiler welfare laws on unit cost. Scientific Papers, Series D. Animal Science 57, 212–215.

[vms372-bib-0048] Tuyttens F. , Vanhonacker F. & Verbeke W. (2014) Broiler production in Flanders, Belgium: current situation and producers’ opinions about animal welfare. World's Poultry Science Journal 70, 343–354.

[vms372-bib-0049] Tuyttens F.A.M. , Federici J.F. , Vanderhasselt R.F. , Goethals K. , Duchateau L. , Sans E.C.O. & Molento C.F.M. (2015) Assessment of welfare of Brazilian and Belgian broiler flocks using the Welfare Quality protocol. Poultry Science 94, 1758–1766. 10.3382/ps/pev167 26049803

[vms372-bib-0050] Vučemilo M. , Matković K. , Stoković I. , Kovačević S. & Benić M. (2012) Welfare assessment of dairy cows housed in a tie‐stall system (in Croatia). Mljekarstvo 62, 62–67.

[vms372-bib-0051] Welfare Quality® (2009a) Welfare Quality® Assessment Protocol for Cattle. Welfare Quality® Consortium, Lelystad: The Netherlands.

[vms372-bib-0052] Welfare Quality® (2009b) Welfare Quality® Assessment Protocol for Pigs. Welfare Quality® Consortium, Lelystad: The Netherlands.

[vms372-bib-0053] Welfare Quality® (2009c) Welfare Quality® Assessment Protocol for Poultry. Welfare Quality® Consortium, Lelystad: The Netherlands.

[vms372-bib-0054] Wellbrock W. , Oosting S.J. , Bock B. , Antunovic B. & Kralik G. (2009) Harmonization of welfare standards for the protection of pigs with the EU rules: the case of Croatia. Italian Journal of Animal Science 8 (Suppl. 3), 21–38.

